# “Like Pieces in a Puzzle”: Online Sacred Harp Singing During the COVID-19 Pandemic

**DOI:** 10.3389/fpsyg.2021.627038

**Published:** 2021-03-19

**Authors:** Esther M. Morgan-Ellis

**Affiliations:** Music Department, University of North Georgia, Dahlonega, GA, United States

**Keywords:** community singing, virtual community, COVID-19, participatory music-making, memory, ritual, Sacred Harp singing

## Abstract

Sacred Harp singers the world over gather weekly to sing out of *The Sacred Harp*, a collection of shape-note songs first published in 1844. Their tradition is highly ritualized, and it plays an important role in the lives of many participants. Following the implementation of lockdown protocols to combat the COVID-19 pandemic, groups of Sacred Harp singers quickly and independently devised a variety of means by which to sing together online using Zoom (“zinging”), Jamulus (“jamzinging”), and Facebook Live (“stringing”). The rapidity and creativity with which Sacred Harp singers developed ways to sustain their activities attests to the strength and significance of this community of practice, and in this article I describe each modality and provide an account of how it came to be developed and widely used. As a participant-observer, I completed extensive fieldwork across these digital sites and conducted semi-structured interviews with 22 other singers. I found that online singing practices have reshaped the Sacred Harp community. Many singers who did not previously have the opportunity to participate now have access, while others have lost access due to technological barriers or lack of interest in online activities. At the same time, geographical barriers have disintegrated, and singing organizers must make an effort to maintain local identity. A stable community of singers has emerged in the digital realm, but it is by no means identical to the community that predated the pandemic. I also identify the ways in which online singing has proven meaningful to participants by providing continuity in their personal and communal practice. Specifically, online singing allows participants to access and celebrate their collective memories of the Sacred Harp community, carry out significant rituals, and continue to grow as singers. While no single modality replicates the complete Sacred Harp singing experience, together they function “like pieces in a puzzle” (as one singer put it), allowing individual participants to access many of the elements of Sacred Harp singing that are most meaningful to them.

## Introduction

In early 2020, social distancing measures were enacted on a global scale to combat the spread of COVID-19 (Lewnard and Lo, 2020). These measures, which prohibited or limited in-person gatherings and interactions, impacted communities of all types. As early as March 2020, however, singing communities had become the focus of special attention. Choir practices in the United States, France, Germany, and the Netherlands were identified as “super spreader events,” and it was suggested that singing might be a particularly effective means of passing on the virus (Bahl et al., 2020; Charlotte, 2020; Hamner et al., 2020, p. 606). Subsequent research focused on the role of aerosols in transmitting COVID-19, with a special focus on aerosol production related to singing (Alsved et al., 2020; Bahl et al., 2020; Becher et al., 2020; Kohanski et al., 2020, p. 1,175). Participants in singing communities were quick to pioneer new ways of carrying on with their activities. Some solutions – such as meeting outdoors, wearing masks, distancing, and employing amplification – have arguably made in-person singing reasonably safe (Nix et al., 2020). Early in the pandemic, however, virtual solutions were necessitated by prohibitions on in-person gatherings, and they have continued to flourish as singers – especially those who belong to at-risk groups – hesitate to return to in-person activity. Virtual singing, however, is made difficult by the fact that an accessible platform for synchronous online singing does not currently exist (Bendall, 2020, p. 70). As a result, communities have had to improvise new ways of coming together in song. The global community of Sacred Harp singers has produced a particularly diverse, creative, and successful set of methods.

**Figure 1:**
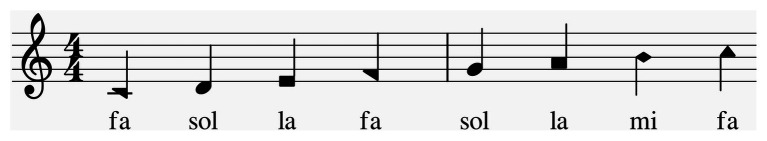
The four-shape system uses four solfege syllables and their accompanying shapes to indicate the seven scale degrees. This system is used in *The Sacred Harp*, *The Shenandoah Harmony*, and *The Southern Harmony*.

**Figure 2:**
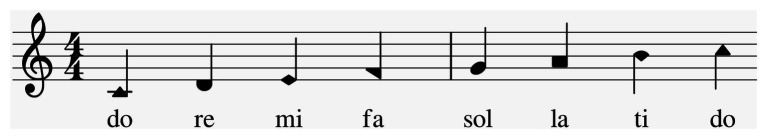
The seven-shape system uses seven solfege syllables (one for each scale degree) and their accompanying shapes. This system is used in *The Christian Harmony*.

Sacred Harp singers gather together to sing primarily out of *The Sacred Harp*, a shape-note song book first published in Georgia (United States) in 1844 (Cobb, 1978, p. 4). In the shape-note tradition, different scale degrees are indicated by variously-shaped note heads (Bealle, 1997, p. 1), and many Sacred Harp singers enjoy singing out of a variety of shape-note books. *The Sacred Harp*, *The Shenandoah Harmony*, and *The Southern Harmony* are four-shape books, meaning that they employ notation in which four shapes designate the seven scale degrees, while *The Christian Harmony* is a seven-shape book (Goff, 2002, p. 24); all four books were used by singers in this study (Figures 1, 2). Participants typically attend weekly practice singings in their local communities and travel to attend all-day “annual” singings and 2- or 3-day conventions, which are somewhat more formal (Miller, 2008, p. 49). There is never a designated director, and all participants are invited to select and lead songs, which are sung through once using the shape names (fa, sol, la, and mi) before being sung on the words. Participants lead from the center of the “hollow square,” which is formed by rows of chairs arranged to face a central point. Although the four sections – treble, alto, tenor, and bass – reflect standard choral practice, it is typical for men to sing the treble and women to sing the tenor, each in their own octave, and the tenor part always holds the melody (Cobb, 1978, p. 8–9). Both leaders and singers beat time, and the style of singing is remarkable for its unrelenting force and volume. Other rituals associated with Sacred Harp singing, of which there are many, will be introduced and considered in the course of this study.

Sacred Harp singers have traditionally been assigned to the categories of “Southern” (rural, Christian, born into the tradition, and working-class) and “Northern” (urban, religiously diverse, came to the tradition as adults, and middle-class). This reflects the history of the practice, which has roots in the South but spread throughout the United States and abroad during the course of a late 20th-century revival (Marini, 2003, p. 82; Clawson, 2011, p. 7). Currently, there are active communities of singers in Australia, Canada, Germany, Ireland, Japan, and the United Kingdom (Caudle, 2021). Although Sacred Harp songs are religious, and singings often begin and end with prayer, the community is diverse in terms of faith. Revivalist singers in particular are likely to be non-believers or to adhere to non-Christian faiths, and singings are always non-denominational. The song texts, therefore, are interpreted on an individual level, and participants are often drawn into the community for reasons that have nothing to do with spirituality, Christian or otherwise.

This study seeks to explain why some Sacred Harp singers continued to find collective singing to be meaningful even under unfavorable online circumstances. To understand how these singers were able to use online participatory activity to sustain their community and reinforce interpersonal bonds, however, we must first address the power of participatory singing to create and strengthen in-person community. Some of the earliest theories about the power of group singing were proposed by sociologists (Durkheim, 1912/1995, p. 232; Collins, 2004, p. 61). Recent empirical studies have revealed that participatory singing does in fact create and reinforce community bonds (Li and Southcott, 2012, p. 67–70; Tarr et al., 2014; Balsnes and Jansson, 2015, p. 168; Pearce et al., 2016, p. 12–15). At the core of this research is the hypothesis that synchronization of voluntary and involuntary biological processes – a process termed “entrainment” (Hayward, 2014, p. 10; Thaut et al., 2015) – can promote altruistic behavior (Kokal et al., 2011; Cirelli et al., 2014), increase positive feelings felt by participants toward one another (Launay et al., 2014), enhance feelings of social connection and inclusion (Lumsden et al., 2014; Welch et al., 2014), improve cooperation (Valdesolo et al., 2010; Reddish et al., 2013), and raise trust levels (Launay et al., 2014). Researchers have demonstrated that synchronous music-making wields greater power than other activities. This is due in part to the effect of “shared intentionality,” which applies to situations in which participants occupy mutually understood roles in the pursuit of common goals (Tomasello et al., 2005; Norton, 2016, p. 66–67).

While all forms of coordinated musical production can enforce social bonds, singing seems to have the most powerful effect. Recent studies have revealed that participation in a singing group raises levels of oxytocin, a hormone known to play a key role in social bonding (Kreutz, 2014; Keeler et al., 2015). It appears that singing is ideally suited to inducing a relaxed and non-threatened state, with oxytocin as one of the contributing factors, and it has been proposed that human beings must achieve such a state in order to engage in social bonding (Vickhoff et al., 2013). Finally, a 2016 study not only replicated previous results by demonstrating that participation in a choir increases feelings of inclusion and connectivity but also demonstrated that these results are intensified when the choir is larger, thereby suggesting that singing is a particularly useful tool for supporting social connectivity on a grand scale (Weinstein et al., 2016, p. 156). Sacred Harp singers are in part rewarded by these psychological and physiological benefits, and I found that singers were often hoping to recreate these experiences by participating in online singing.

## Study Approach

The object of this ethnographic investigation was to document the practices and motivations of online Sacred Harp singers and to understand how online singing has served both to sustain and reshape Sacred Harp communities at the local and global levels. Ethnographic methodology combines participation, observation, and questioning to gain insight into the complex workings of individual communities (O’Reilly, 2012, p. 1). It does not provide quantitative answers to focused research questions, but instead seeks “thick” descriptions of social phenomena (Geertz, 2004, p. 145–146). This study, therefore, is wide-ranging, for it reflects broad yet intensive engagement with diverse online singing practices. In this article I will detail those practices and interpret their social and psychological functions. However, these results do not transfer easily to other singing communities, and some observations could be strengthened by the application of quantitative methods.

This is a study of a community of practice. Communities of practice have been defined as “groups of people who share a concern or a passion for something they do and learn how to do it better as they interact regularly” (Wenger-Trayner and Wenger-Trayner, 2015, p. 1). All members of a community of practice are “legitimate participants” (Blanton, 2016, p. 7), and Sacred Harp singers eagerly welcome new members into their ranks. However, “peripheral” participants lack the extensive knowledge, skill, and social connections of “full” participants, who have participated for many years and occupy honored roles (Lave and Wenger, 1991, p. 37; Morgan-Ellis, 2019, p. 31–32).

The collective of singers who participate in online activity also satisfies accepted definitions of “virtual community” (Ridings and Gefen, 2004). Virtual communities of music practitioners (Bryant, 1995; Waldron and Veblen, 2008; Waldron, 2009, 2011, 2013) and fans (Watson, 1997; Kibby, 2000) have been the topic of fascinating research, as has the role of virtual community in sustaining cultural and interpersonal ties in diaspora (Wenjing, 2005), a framework that has been applied to the Sacred Harp community (Miller, 2008, p. 28). This virtual community differs from others, however, in that it is expected to be temporary. Most of its members are seeking not to replace or augment in-person experiences, but rather to sustain their communities and practices through a period of social isolation that they know will come to an end. This virtual community also differs from those studied in the past insofar as its focus is on active, synchronous participatory music-making as opposed to discussion, networking, the sharing of information, or pedagogy. While other virtual music communities have supplemented or supported individual musical practice (whether in isolation or as part of a local in-person community), online Sacred Harp singers seek to synchronously recreate their in-person experiences with maximum fidelity. The ways in which they have done so are novel and diverse.

### Summary of Fieldwork

I began participating in online Sacred Harp singing on April 10, 2020 (Morgan-Ellis, 2021b). I took notes on my experiences from the start, eventually accumulating nearly 6,000 words in reflections on various online singing experiences (Stock, 2004, p. 23). For the next 3 months I remained heavily involved in Facebook-based singing (“stringing”) and carried on regular correspondence with other participants. On July 19, I posted to the Sacred Harp Streaming Facebook group to ask if anyone would be interested in having a conversation. I received a strong response, and between July 20 and September 11 I was able to conduct semi-structured interviews over Zoom with 22 singers (Dunn, 2005, p. 80). Although I prepared a list of questions in advance of the first conversation, my focus quickly shifted as I learned more about the activities and experiences of other online singers. I always allowed my interlocutors to guide the conversations, which were between 15 min and 2 h in length, with an average duration of about 50 min. Although my approach to recruiting interlocutors produced a far-from-random pool, the singers with whom I spoke represented the diversity of the community. They had broad experience with the modalities described below: 13 engaged regularly with zinging, two with jamzinging, and 17 with stringing, while three had participated in virtual choirs. They belonged to all adult age groups, had been singing for periods of time spanning 1 month to 60-plus years, and resided in 14 of the 50 states that comprise the United States and in Scotland. The vast majority might be classified as revivalists, but two were raised in the singing community and can be regarded as tradition-bearers. In discussing the development and operation of online Sacred Harp singing, I will identify interlocutors who occupied public roles by name. In sharing personal responses to online singing experiences, I will identify interlocutors only with relevant details concerning their location or experience (following Miller, 2008).

These conversations transformed my own experience with online singing. To begin with, I learned about the existence of various Zoom-based singings (“zingings”) and began attending on a regular basis. One of my interlocutors added me to a Facebook Messenger group that he used to announce singings on a near-daily basis, while another recommended that I follow her lead in joining all of the Sacred Harp groups on Facebook. In particular, I became a regular participant in the Utah (Salt Lake City) and Twin Cities zingings, at which I regularly encountered my new friends. I also attended zingings in Toronto, Seattle, Portland, Philadelphia, and Savannah, and participated in several annual events. My conversations with singers changed my own thinking about online Sacred Harp singing in ways that are so profound that I can no longer extricate my own ideas from those of my interlocutors. While I will quote their words throughout this discussion, the experiences and perceptions they shared with me came to shape all aspects of this account.

## Results and Discussion

### The Architectures of Online Sacred Harp Singing

Perhaps the most extraordinary aspect of Sacred Harp singing during the COVID-19 pandemic is that so many members of the community quickly and independently developed means by which to continue singing together. In many cases, a platform that was never intended to facilitate participatory music-making was creatively adapted. The drive to sing together proved so strong that many local Sacred Harp communities were gathering and singing in virtual spaces within a week or so of quarantine being imposed. Here, I will outline the ways in which various technologies have been used, provide accounts of how they came to be adopted by different communities, and reflect on the strengths and weaknesses of each approach. One Minnesota singer with whom I spoke described online singing modalities as being “like pieces in a puzzle”: No single modality replicated the in-person experience, but together they largely fulfilled her needs. Most of my interlocutors celebrated the fact that different singers preferred different online solutions, but each also had their own strong opinions.

#### Zinging

The most common forms of online singing activity use Zoom as a platform. (Such gatherings have been affectionately nicknamed “zingings” by California singer Pat Coghlan, whose terms I have adopted for the first three modalities.) Zingings are hosted by local singing communities and usually take place at whatever time in the week that community habitually gathers. However, because geographical location no longer bars attendance, most communities have opened their zingings to visitors. Some zingings are announced on a public calendar put together by Utah singer Evelyn Lamb, while others are advertised informally in Facebook groups. Various annual singings and conventions have also been reimagined as zingings.

Under the header of zinging, however, fall two distinct activities. Zoom cannot facilitate synchronous participation, so it has been necessary for singers to develop alternative approaches. In some cases, a host shares video and/or audio of a song – usually captured at a live event – and participants sing along on mute. In others, individual participants take turns unmuting to lead a song. Often, leaders are encouraged to sing the tenor, although singing other parts is generally tolerated. As with the recorded media, other participants mute themselves and sing along. Some zingings rely entirely on recordings, some entirely on live leading, and some on a mix of the two.

There are various ways in which hosts of zingings that rely on recorded media (often referred to as DJs) can operate such gatherings. Lamb, who hosts the Utah zingings, usually shares audio and video of live recordings. Participants can request songs in advance or during the zinging. If a participant has a certain video they want to see they can share the link, but if not she will find something on the spot. For each song, Lamb invites the person who made the request to unmute and say something about their choice. On weeks when the Utah singers use the 2012 Cooper revision of *The Sacred Harp* (known as the Cooper book), Lamb will share the notation so that participants who do not own the book can still join in. Lamb developed her approach by experimenting with other Utah singers in the 2nd week of quarantine, meaning that the full group was able to come together on Zoom after missing only a single scheduled gathering. It seems that other groups independently arrived at similar solutions, although there is variation. For the Toronto *Shenandoah Harmony* zingings, for example, the DJ assembles a YouTube playlist ahead of time and allows three songs to play without interruption, pausing between sets for conversation. When that group sings from the 1991 Denson revision of *The Sacred Harp* (known as the Denson book), three couples take turns live-leading for the 1st hour, while a YouTube playlist fills any time that remains after a social break. In Seattle, a team of three facilitators share the duties of engaging verbally with participants, finding recordings to fill requests, and broadcasting those recordings. The Twin Cities zingings employ live-leading for bandwidth reasons; participants found that sharing YouTube videos over Zoom was not feasible.

Many of the singers with whom I spoke derived a great deal of satisfaction from video-based zingings, the reasons for which are addressed below. Others, however, could not see the appeal. One Kansas-based singer described the experience as “musically deeply unsatisfying” and indicated that she would prefer to sing along with recordings in isolation. Many singers missed the “surround sound” effect of in-person Sacred Harp singing, during which the leader is buffeted by sound from all directions. Even the best recording cannot come close to replicating this experience. Many also shared my observation that the sound quality of videos – most of which had been captured by amateurs and already lacked fidelity – was often unsatisfactory after having been broadcast through Zoom.

The issue of “liveness” arose in many of my conversations with singers who had participated in video-based zingings. For some, the fact that videos captured a past event prevented them from satisfactorily standing in for a real-time experience. “Because it’s a recording,” one California singer told me, “and you know it’s a recording, it’s just not the same.” For this reason, she preferred stringing. A Minnesota singer mourned both the responsiveness of live singing, which is lost with the use of recordings, and the fact that Sacred Harp singings are by their nature “live and real and not repeatable. Each one is a little journey.” This attitude was not universal; when I asked a Tennessee singer whether the fact that videos were not “live” explained her distaste for zinging, she dismissed the idea: “Oh see, that does not affect me. That does not bother me.” Most singers, however, valued the responsiveness of live singing partners and the unpredictability of an in-person singing, which always carries the risk of false entrances, wrong notes, and even total disintegration. Sacred Harp singers also carry memories and stories of unique singing experiences – gatherings where something indescribable and unrepeatable happened during the music-making. Experiences like these, which are much valued, cannot result from singing along with a recording.

Those who enjoyed zinging usually described how gratifying it was to see the faces of other singers. One Tennessee singer even pointed out that Zoom is superior to in-person singing in this respect because the participant gets to see everyone head-on, which is not possible in the traditional Sacred Harp singing formation. (The same singer has taken to hiding her own video because she finds it distracting.) Although a shared video automatically dominates the screen in Zoom, the individual user can choose instead to look at the faces of individual participants, and many singers do so. “I usually drag my screen over to where I’m seeing everybody,” one Alabama singer told me, “because I want to see the people.” The same singer also told me how rewarding it was to look up and see the other participants while singing a familiar song. A California singer took the same approach, “Because the best way of Zoom singing – zinging – is where you are looking at all the people. You get a better sense of community, and a sense that you are singing with other people.” I frequently did the same myself, expanding my Zoom gallery to cover the video and looking up whenever possible. Some participants, however, enjoyed watching the videos for reasons that I will explore in a later section.

#### Jamzinging

Some singers have taken to using Jamulus, which is a computer program that facilitates low-latency audio communication. This allows them to sing together in real time. While there are regular Jamulus singings in the Bay Area, Omaha, and Boulder, the group in Philadelphia pioneered the use of Jamulus in conjunction with Zoom. The host mixes and shares the audio output from Jamulus, while Zoom-only participants are welcome to sing along on mute. During breaks, the Jamulus audio is disconnected and Zoom participants are able to speak with one another. Sometimes the host also broadcasts the sessions over Facebook Live, making participation available to even more singers.

The obvious advantage to using Jamulus or another low-latency program is that participants get to engage in a multi-directional singing experience, in which they can both hear and be heard by the other singers. However, low-latency technology is still developing, and it presents certain challenges. To begin with, the sound quality is not always good. In notes from my visits to Philadelphia jamzingings I described it as “tinny” and “crackly” and recorded problems with balance and microphone peaking. One singer described the sound as “distressing,” while another found that the volume was too low to facilitate participation. “I’ve just about had to whisper sing,” she told me, “because if I sang normally I would not be able to hear.” In addition, some participants experience significant lag, either because they have a slow connection or are too far away from the server. The host can reduce the volume of these singers so that the delay in their channels does not disrupt the overall experience. Adjusting the balance is a tricky process, however, and technological concerns can take up a considerable amount of time, such that we only sang four songs in the first 30 min of one July meeting. Many of these challenges were overcome in the ensuing months as the singers mastered the technology.

At the time I completed interviews, few of my informants had used Jamulus. A Maryland singer who reported good Jamulus experiences in general described the jamzingings as “sometimes great, and sometimes weird and laggy and really frustrating.” On these less successful occasions, she found that “it feels like you are really not singing, and it feels so isolated and terrible.” Joining a jamzinging *via* Zoom only constitutes a different experience. There is a clear division between the audible Jamulus singers, who are also able to chat and who get to call songs first, and the inaudible Zoom singers, who are inevitably sidelined. My Maryland informant, who usually sings over Jamulus, joined the Philadelphia jamzinging *via* Zoom once and was frustrated by her inability to comment on the proceedings, while my Scotland informant, who at first joined only *via* Zoom, shared my own feeling of superfluity. When she was later able to join *via* Jamulus, however, “I got three bars in and then cried for the next 2 h” – a response that I know was shared by many other users.

#### Stringing

The most unlikely form of online singing is stringing, which uses Facebook Live in a manner that was never intended by the architects of the social media platform. In stringing, a participant broadcasts themself singing along with another Facebook Live broadcast in order to add a part to the texture through a process of live overdubbing. Stringing was pioneered quite accidentally by Emily Ross in March of 2020. Two friends of hers, David Olson and Peter Stenshoel, were broadcasting themselves singing the bass and tenor parts of Sacred Harp songs over Facebook Live. Ross was singing along and wanted to take a video of herself to send to them privately. Because her phone was not handy, however, she opened an additional browser tab and began to stream her own singing. She alerted another friend, Irene Gilb, who did the same, thereby adding the fourth part. Following a couple of weeks of experimentation, Gilb created a tutorial video explaining the procedure and founded the Facebook group Sacred Harp Streaming for the purpose of drawing attention to the practice and organizing online singings (Gilb, 2020).

Stringing can take many forms. Because any number of people can broadcast themselves singing along with each individual stream, participation can branch almost indefinitely. The length of any given chain is limited only by the loss in sound quality that occurs when a broadcast captures the audio emanating from the speakers. Some deterioration is inevitable, but it can be minimized with good equipment (Morgan-Ellis, 2021b). Naturally, singers can join in without broadcasting themselves, which has proven to be the typical mode of participation. The participant experience is synchronous; that is to say, each singer is able to synchronize perfectly with the streaming audio. In real time, there is a delay of about 30 s between sending and receiving *via* Facebook Live, but because the broadcast is unidirectional this is of little consequence. Participants are able to interact with broadcasters by leaving comments, while broadcasters can either speak directly to their viewers or type responses.

Stringing has much in common with live-led zinging, insofar as a “muted” participant is able to sing along with a live leader. The disadvantage is that the leader cannot see the other participants, and participants cannot see or speak to one another (although they can communicate in the comments). The advantages, however, are several. To begin with, stringing make available the option of adding parts until the texture is complete. In addition, the sound quality over Facebook Live is generally superior to Zoom. Also significant is the fact that stringing takes place in an open arena – Facebook – as opposed to a closed meeting. If a participant chooses to stream publicly, hundreds of people might “wander through” the sing. Non-streaming participants can choose whether or not they want to disclose their presence, and they are not subjected to the peer surveillance that is typical of Zoom meetings.

Stringing can be a highly imaginative activity, for the participant must actively conjure the presence of unseen and unheard singers. I discovered that those who found this modality to be rewarding had no trouble feeling as if they were participating in a communal activity. As one California singer who regularly broadcasts herself told me, “I know there’s people there. I’m not necessarily thinking about the anonymous at large, but it’s nice to be thinking about that person that is right there at the time.” A Maryland broadcaster agreed: “I also feel that it’s very real. […] I have the very strong feeling that people are singing with me, especially when they comment, but sometimes just when the name pops up. I’m like aww, that person, yay, you are here with me.” A Pennsylvania singer who does not broadcast described seeing the names of other singers as meaning, “We’re together. We’re in that same space, having the same experience. Even if she does not know that I’m having that experience with her, I am. It’s like being in another dimension.” Although this singer had powerful communal experiences without broadcasting, I found that serving as a link in the chain caused me to feel more deeply involved than did simply singing along, although both were meaningful. At the same time, broadcasting brought risk. I experienced the greatest feelings of connectivity and communality when I knew that followers were singing with me, but also feelings of overwhelming loneliness when I was not aware of followers, either because they were refraining from commenting or simply were not there. The relative “size” of a stringing – which I was aware of through viewer counts, comments, and notifications – greatly impacted the experience. A stringing with lots of text-based interaction, in which I knew that dozens of people were participating, felt busy and social, while one with only a few participants felt physically small (Morgan-Ellis, 2021b). “It’s so intimate sometimes,” agreed the same Maryland singer. “It’s really intense.” Our shared response was determined not by what we heard or how we sang, but by the ways in which we imagined the singing community.

Broadcasting, by its very nature, can transform a participatory act into a presentational one (Turino, 2008, p. 26). A New Mexico singer commented on how she makes an effort to avoid thinking or behaving as if she is performing for an audience, but sometimes finds it irresistible. One of the singers who frequently originates streams told me flat out, “It’s like a performance for me,” noting that a 2-h stringing always leaves him exhausted. He later nuanced his perspective, describing his efforts to stay in a participatory “mindset” and reflecting on how the feeling of broadcasting changes over the course of the 2 h.

The fact that Facebook Live broadcasts are automatically preserved (the user has to actively delete the video to remove it from their timeline) further transforms the experience of stringing, for most broadcasters are aware to varying degrees that they are producing a lasting audiovisual product. This has its benefits: One California singer who broadcasts, for example, likes to visit other streams after the singing has concluded to read comments and even sing along. Although he sometimes feels like he’s “missing out on things” while broadcasting, visiting other streams after the fact allows him to “get the sense of community.” A Texas singer often sings with old streams, although he appreciates the fact that people can choose to participate live.

“Liveness” was generally important to members of the stringing community. For the most part, they valued the fact that singing took place essentially in real time. A New Mexico singer reflected on this element at considerable length: “Knowing that you could say something to anybody along the way, and they would get it not exactly in real time but pretty darn close, I think that that is a key factor to all of this because […] one of the things that is important in Sacred Harp, whether people know it or not, is that they are being heard, and they are hearing others.” Not all of those who participate in stringing can be heard in the conventional sense, but they can all express their thoughts and feelings in ways that will be “heard” by other participants while the singing is in progress.

I spoke with several singers who had tried stringing and did not find it appealing. Most of the reasons had to do with what one Washington singer described as the “tunnel-visioned” nature of stringing, which limits both the visual and communicative elements of the participatory experience. That singer was most frustrated by the delay in feedback and the lack of certainly that anyone was singing with him. Some wanted to hear all four parts; “I do not really want to do a duet with one person,” one singer remarked. Some missed the socialization of zingings, and felt that streamers were too focused on getting through lots of songs. A California singer who enjoys all modalities summed up what many seemed to feel: “Stream singing is better for the music, but for community, Zoom singing has it.”

Several of my interlocutors compared stringing to a small-group singing. “It’s like sitting in a singing with just the four of us,” commented the same California singer. The Maryland singer proclaimed that “in some cases, it’s better than small singings.” She described how the social dynamics at house singings can sometimes make the experience unpleasant and leave her feeling bad. “But I feel pretty good after all of the streaming singings. […] In terms of mood lift, it’s pretty close, or better sometimes.”

#### Virtual Choirs

Although virtual choirs are not a focus of this study, they must be addressed. A virtual choir is a video or audio presentation assembled out of recorded contributions from individual singers (Carvalho and Goodyear, 2014, p. 212; Fancourt and Steptoe, 2019; Bendall, 2020, p. 71). Virtual choirs exploded in popularity following the onset of the pandemic as ensembles and singers searched for ways to collaborate and present their work, and it is natural enough that Sacred Harp singers were among the many to pursue this approach (Morgan-Ellis, 2021a). One notable project was the 331-singer performance of *Easter Anthem*, published to YouTube on April 12, 2020 (Taylor et al., 2020). Several of my interlocutors also participated in Dispersed Harmonies, a semi-stable group of singers who took turns “calling” songs by filming themselves pitching and singing a selection. Other singers were then able to listen to the lead (as in stringing or live-led zinging) and film themselves adding a part. At the end of the process, a volunteer would assemble the videos into a virtual choir and publish the result to YouTube (Geerts, 2020).

Virtual choirs contributed a significant piece to the singing “puzzle,” and many singers found their participation meaningful. One Seattle-based member of Dispersed Harmonies told me that “even though it’s not real time, it’s like I’m singing together with these people,” confirming that virtual choir participation can indeed constitute a communal experience for some. Another singer likewise described the experience as “singing to and with another person,” and also commented on the communal “trust and faith” involved in the sharing of individual videos. She connected the experience with sitting next to a singer who may or may not be good, and observed that both in-person singing and participation in Dispersed Harmonies carry “a certain responsibility in respecting people and their individualities.” A Minnesota singer shared the gratification of “lending to make that sound. […] That part of contributing that felt really good.” All the same, the Seattle singer acknowledged that the product-orientation of virtual choirs is at odds with Sacred Harp singing. “Up until now,” he reflected, “it’s never been about a product you have at the end. It’s just about doing it while you are doing it, singing together. So changing that orientation, it’s never the same.”

On several occasions, virtual choir recordings were produced explicitly for use in a zinging. One of my interlocutors called a song with Dispersed Harmonies so that the video could be used to close a zinging hosted by the group in Scotland. The virtual choir included participants from the local community. The co-chairs of the Minnesota Convention created virtual choir videos for the opening and closing songs that featured their two faces and the voices of several other Minnesota singers. And I myself participated in an audio-only virtual choir put together by Lamb, who wanted to fulfill a request for a song from the Cooper book but was unable to find a recording. She later played the assembled performance at a Utah zinging for participants to sing along with, and also published it to SoundCloud so that other DJs could use it for the same purpose (Lamb, 2020).

### The Geography of Online Sacred Harp Singing

Sacred Harp community has always encompassed both global and local identities. All singers belong to a unified, global community – referred to by Miller as the Sacred Harp diaspora (2008, p. 28) – and they can be assured of a welcome at any Sacred Harp gathering (Lueck, 2015, p. 124). The rituals of Sacred Harp facilitate easy assimilation into a new group of singers. Many of the most dedicated singers “travel,” meaning that they make frequent trips, often over great distances, to attend conventions and all-day singings. In doing so, they forge relationships with singers who belong to other localized communities and reinforce the idea that Sacred Harp transcends geographical bounds. At the same time, local communities are important. Singers often care deeply about the individuals with whom they gather on a weekly basis, and local groups often develop rituals of their own. In addition, many singers do not travel, and as a result might feel more deeply attached to their local community than to the imagined global community.

In many ways, the online Sacred Harp landscape has maintained these traditional elements. Each regular singing – whether a geographically-anchored zinging or a relatively “placeless” stringing or virtual choir – constitutes a “local” community, and each online singer has found a home community (or several) in which they regularly participate. Online singing, however, has reshaped the Sacred Harp community at both the global and local levels by simultaneously increasing access for some and diminishing access for others. Online singing also all but eliminates geographical barriers to participation. As a result, a new geography of Sacred Harp singing has emerged.

#### Reshaping the Community

The community of online singers is not identical to the community of singers who were active before the pandemic. On the one hand, many avid singers have not taken to online singing. As one Minnesota singer put it, “Our community continues, but it’s just a subset.” It has been well documented that individuals experience their participation in virtual communities differently, and these variations certainly contribute to the diversity in interest levels (Blanchard and Markus, 2004, p. 71–73). The reasons for which Sacred Harp singers did not engage with online activities varied widely. Many of my interlocutors reported that some community members had joined a zinging once and then disappeared with comments that the activity was not interesting to them. One of the zinging facilitators with whom I spoke confessed that he finds fulfillment in bringing the community together but does not enjoy the singing; this was due to the fact that he lives in an apartment and cannot sing at full volume, as is typical of in-person gatherings.

Some singers were not able to find a sense of community connection *via* online modalities. Many of the singers with whom I spoke had lengthy experience with online community and were comfortable with the idea of making remote connections; as a result, they had no trouble translating their Sacred Harp activities to the digital realm. Some, however, did experience feelings of isolation associated with online singing. As one singer told me, “Sometimes I find it comforting and useful. Other times I feel more lonely.” It seems certain that many singers – especially those without experience participating in online communities – must have experienced *only* the isolating aspect of online singing and therefore taken no interest in the activity. It is also certain that some singers do not regard online singing as “real,” per comments made on the Sacred Harp Streaming Facebook page and elsewhere on social media.

Other singers might find the activities rewarding, but lack the necessary equipment or Internet access or are simply unaware that online singing is taking place. To engage with online singing requires, at the very least, a decent speaker and a reliable Internet connection. I heard reports of singers whose connections were so poor that participation was not rewarding. I myself struggled to manage stringing, which requires significant bandwidth due to the simultaneous receiving and broadcasting of streams, and I suffered through various difficult Zoom sessions in which live leaders froze or had inconsistent audio (the platform, which is naturally geared toward speech, automatically slows and accelerates audio if the connection is poor). Zinging is certainly more rewarding with a large screen, while broadcasting over Facebook Live works best with two devices and a good microphone. Many singers reported frustration with the limitations of their technology. Other singers reported Zoom fatigue: After spending all day on the platform for work, they did not want to log on to yet another meeting as a form of recreation. Further study is needed, however, to understand the many reasons for which some Sacred Harp singers found online singing to be unsatisfactory.

On the other hand, online singing has attracted participants who did not previously have access. While most of my interlocutors belonged to local singing groups, three had no opportunities to engage regularly in Sacred Harp singing before the pandemic and were enthusiastic to reconnect with distant singing friends online. Two had moved away from active singing communities and were able to reengage with singers they had left behind. The third, like me, lives in an area that has no Sacred Harp activity. She traveled a lot, however, and has been able to reconnect with friends whom she now sees online; indeed, at the point I interviewed her on July 20 she had already participated in 70 zingings, and I saw her at many more over the next few months. I had few friends in the Sacred Harp community before the pandemic, but always enjoyed singing when the opportunity arose. As I became involved with online singing, I made significant new social connections (Morgan-Ellis, 2021b).

As a result of these shifts, the online singing community seems unfamiliar to some. A longtime singer explained to me why she has little interest in online singing: “I have very strong personal connections in the singing community, and most of the people that I am very close with have not been involved in singing online at all. And therefore it’s like watching this whole different group of people assemble and suddenly I do not know any of these people. So I probably would be more interested in singing online regularly if more of my pre-existing singing friends were involved.” She was pleased to hear about the positive experiences that others were having, and interested to see the emergence of what she described as a “parallel community,” but she did not feel that she was a part of that community.

At the same time, other singers reported forging new relationships through their online activities. “I’m really enjoying people I’m meeting,” a Minnesota singer told me. “I meet people from all over the world.” One California singer likened the experience to attending an in-person event: “If you go to a convention you are going to meet someone that you have not known before.” For him, making new connections is part of Sacred Harp participation, and he enjoyed the opportunity to meet people online. Actually singing with others – even virtually – is an important part of building relationships in the Sacred Harp community. One Tennessee singer reported her delight at having “sung with singers that I have not sung with before,” even though they could not hear one another’s voices.

The nature of the online modalities requires individuals to assume leadership roles that did not previously exist. The facilitator roles described in the context of video-based zinging and jamzinging have no precedent, and these individuals exercise an outsized role in shaping the singing experience. An in-person weekly singing is not overseen by any one person, and even an annual singing, in which individuals occupy named roles and an organizing committee determines the sequence of leaders, is principally a collective affair, with no single personality at the center. A large Zoom meeting, however, requires a host, whose roles include calling on other to speak, muting and unmuting participants, and even determining who can enter or stay in the meeting. Jamulus – at least when used in conjunction with Zoom – requires that a single operator adjust the balance of participants and possibly mute those who are having problems with sound quality or lag. Similarly, stringing brings those who broadcast to the forefront. One participant expressed concern to me that stringing introduces inequality into what should be a fully egalitarian community. “Some people are being known now more than others,” she lamented, observing that both broadcasters and frequent commentors were becoming more familiar than other participants. As she summed it up, “There’s more of a chance for people to feel that they are less.” Only the Twin Cities zingings replicated the traditional decentralized structure. In these zingings, the host served a purely technical role (e.g., typing song numbers into the chat) and participants unmuted themselves at will to lead songs. However, no other group adopted this approach, and centralized models were clearly more popular. The decentralized approach worked best with small groups, eliminated the possibility of video use, and often resulted in long periods of silence between songs – all possible reasons for its limited use.

Almost all of my interlocutors expressed deep concern for those who did not participate in online singing. “I am especially sad for people who do not have the technology,” remarked the same Tennessee singer, “or even the mental experience and knowledge to use technology that might be available in their home or area, as well as the folks who do not have rural broadband access. […] I know that this is a very lonely time.” A Minnesota singer echoed her concern: “I really worry about people who do not have technology period, or if they do they just do not know how to do it, or they are not even Facebook users and they do not even know what they are missing.”

At the same time, the fact that online singing can grant access to previously excluded community members has proved meaningful not just to those singers but to the community as a whole. Many of my interlocutors expressed a hope that some form of online singing would continue into the future. At conventions and all-day singings, participants always take time to name and remember the “sick and shut-in” who are not able to be present (Miller, 2010, p. 253). During the pandemic, however, many of those singers were in fact able to participate, which in turn has produced enthusiasm for the continued use of technology. “I imagine moving forward,” reflected one Kentucky singer, “a possible use of Zoom would be to actually broadcast from live singing for the sick and shut-ins. […] I think it will continue to be important as a way to include people who otherwise could not be included easily.” Lamb told me that her community has considered continuing to stream over Zoom once a month or once a quarter in order to maintain access for singers who cannot attend in person – a practice that some communities had experimented with before the pandemic, but that is now likely to become widespread.

#### Maintaining Local Identity

Taking singing activity online means – at least theoretically – that anyone anywhere can participate in any gathering. This introduces potential for the erasure of local identity. Some geographically-based groups mitigated this by keeping their zingings private, but most sought to retain or create a sense of local identity while opening participation to outsiders. Lamb spoke to me at great length about her efforts to ensure that the Utah zinging continues to feel like “a Utah event.” Although she advertises it widely and welcomes visitors from all over the world, it is important to her to “preserve some amount of feeling like this is our Utah community coming together.” After having visited zingings throughout the United States, I can confirm that each is unique. In some cases, the variations have to do with the interests, personalities, or abilities of the facilitators. In others, they represent continuations of pre-pandemic practice.

To begin with, most groups are meeting at the same times and maintaining the topics and character of their respective singing days. Many groups rotate between shape-note books. In Utah, for example, they sing from the Denson book on the first and second Tuesdays of each month and from the Cooper book on the fourth Tuesday. If there is a 5th Tuesday in the month, they sing from a lesser-known book, such as *The Shenandoah Harmony* or *The Christian Harmony*. They have maintained exactly the same schedule, with one alteration: Instead of taking the 3rd Tuesday off, they hold an additional Denson singing. In the Twin Cities, the Sunday sing is typically more formal while the Tuesday sing “has the feeling of being a little bit looser” – a characteristic, according to one Minnesota singer, that has become even more pronounced in the online environment. Internal rituals are also maintained. For example, Utah zingings always begin with that group’s traditional opening song, while in the Twin Cities, participants stand for the closing song.

Local groups have also developed new rituals. In Seattle, for example, the facilitators integrate Sacred-Harp themed trivia questions and send participants into breakout rooms to chat – strategies that were described to me as providing a “kind of substitute for connecting in the same way that the recordings substitute for singing together.” Lamb’s new practice of asking the person who called a song to explain their choice has certainly become integral to the Utah zinging experience, and was commented on by several of my interlocutors. Participants in the live-led Twin Cities zingings have adopted the practice of praising one another’s solo “performances” – something that would have no place in a traditional singing. However, this behavior is very much in keeping with Sacred Harp values, which emphasize mutual support. One California singer told me about his own experience taking a chance leading at the Twin Cities zinging. Although he was pretty sure he did a poor job, the others praised and encouraged him, and he soon made leading a habit. “That’s what you go to the singing for,” he told me, “to be a witness to and a part of that kind of community.” Each local zinging has developed a distinctive character, and the singers who visit various zingings have unique experiences at each “location.”

In sum, the notion of “traveling” remains nearly as relevant in the world of online singing as it was in the physical world. One zinging organizer used exactly that language when describing the events he organizes: “Every singing that we have is a little bit like these singings at conventions used to be, like, who’s going to show up, who’s going to be the farthest traveler.” Although it has become much easier to travel to a singing, and the fringe social experiences have been eliminated (to the profound regret of many), participants still report a powerful sense of visiting and singing with specific geographic communities. It also seems significant that no generic online singing practices have emerged. Although it would be practical for Sacred Harp singers everywhere to join a unified, place-less zinging, such an event has not once taken place.

### Sustaining Sacred Harp Community and Practice in Online Environments

Most of the singers whom I interviewed found online singing to be profoundly rewarding. Indeed, they generally reported surprise at how meaningful and “real” the experience was. “I would not have expected to tolerate it at all,” one Utah singer told me. “And it’s been surprisingly nice. Surprisingly beyond tolerable, surprisingly actually nice.” The reasons for the success of online singing have to do with the motivations and practices of Sacred Harp singers. All online activities facilitated the continuation of preexisting relationships and communities, and those relationships made online singing meaningful to many participants. If only the relationships mattered, however, Sacred Harp singers might as well have come together in Zoom just to chat (as indeed they did in some places). The question that governed my investigation, therefore, was this: Why sing? Through participation and interviews, I found that communal singing sustained Sacred Harp communities in ways that transcended verbal interaction. I identified three elements in particular that translated well to the available online modalities: the foregrounding of individual and collective memory; the prizing of ritual, which – in combination with memory – serves to sustain community identity; and the valuation of learning and personal growth.

#### Memory

“We have so many memories we can call on,” remarked a participant in a Twin Cities zinging. She seemed to be trying to explain to herself and others why she found the activity we were all engaging in to be so meaningful. The practice of Sacred Harp singing is rooted in memory: both the individual memories that singers carry of past experiences and friends, and the collective memories of the tradition and its most noteworthy bearers (Miller, 2010, p. 259). The significance of memory was borne out by several interviews in which my subjects were loath to discuss their online experiences, but instead wanted only to talk about singers they had known and conventions they had attended. It became clear to me that online singing allowed these individuals to relive those memories. The nature of Sacred Harp singing, which constitutes by the iterative rendition of a bounded repertoire under fixed conditions, encourages the revisiting of memories, as does the tradition of celebrating the deceased with memorial singings and lessons. One California singer described Sacred Harp singing as a séance and told me that community members who have passed on “are always with you when you are singing.” For many participants, therefore, online singing granted access to memories in the same ways as in-person singing, and as a result served the same function in their lives.

Although neither leaders nor participants are traditionally encouraged to talk between songs, every online singing in which I participated was punctuated and shaped by the sharing of memories (Miller, 2010, p. 257). At the Utah zingings, for example, when singers were invited by Lamb to engage in “a little personal sharing,” their remarks almost always related to memory. Sometimes the singer would comment that it was the first song they had ever led, or recall how they had sung it at a specific convention, or associate it with an absent friend. Songs were frequently dedicated to those who had recently died, or to community members who could not be present. At a Twin Cities zinging, a song was sung to honor a convention that most of those present had attended the previous year but that was canceled for 2020. At a Seattle zinging, a participant shared a comment that a famous singer made to her the first time she had led a song, many years before. My notes are full of these incidents, which were so commonplace as to become part of the ritual of the zingings themselves.

Videos and recordings also served to elicit memories. Lamb took great care in selecting recordings, choosing whenever possible a video or audio recording that included people present in the zinging. In the best-case scenario, she was actually able to play a video in which the song was led by the person who called it. This was made possible by the fact that many annual singings are filmed, and many singers travel to be present at such events. The videos themselves meant a lot to some people. “They’re touching,” remarked a lifelong singer who was able to recognize many friends among the faces. Upon playing videos made at a Texas convention, Lamb shared her own memories of learning to sing in that community. In particular, the voice of a singer who regularly followed songs with the remark “good” brought her back to the early days of her participation. Many singers were able to recognize specific recordings or individual voices, even without the aid of video. Familiar singers who were seen or heard in recordings were always named and recalled.

Imagined and remembered voices were as important to the experience as heard voices. In one Twin Cities zinging, a participant commented that she could “hear” a friend who commonly sings an unwritten part to one of the songs – an experience that allowed her to remember and sing the part. A Tennessee singer explained to me how she was able to “hear” the voices of other singers in zingings, even though they were muted. She listed regular participants with whom she has sung in the past and whose voices she knows well, and described how seeing them in Zoom conjured the sounds of their unique voices. For this singer, voices were only one part of a complex of memories associated with each friend that also included their favorite songs and the dishes they bring to gatherings. For her, imagination “fills in all the details” that are missing in the online environment.

Imaginative engagement allowed many singers to feel as if they were “in the Square again” (as one California singer put it), and I heard frequent reference, both in interviews and zingings, to the sensation of “sitting on the bench” in a section. These singers accessed their memories of in-person experiences to fill in the gaps left by online singing. “I think it would be very strange for someone who had never sung before to attend one of these [zingings],” remarked a Seattle singer. “But we kind of all have the details filled in in our own imaginations.” A Minnesota singer made a parallel observation: “You’re summoning up the memory of what it felt like to sing in our usual Tuesday night space, of what it was to sing with a group of people. If you were starting with this you would never get anywhere, but when you have that memory of singing the song in the big room…” Several of my interlocutors made similar references to their habitual singing spaces, which were sometimes visible on-screen either because the broadcast was originating from such a space (e.g., Olson and Stenshoel’s Facebook broadcasts from their living room, where they hosted weekly house singings before the pandemic) or through the use of Zoom backdrops and videos. Often, participants found themselves compelled to execute physical actions – for example, turning to cue the different voice parts – that would have been appropriate in-person but have no meaning in an online environment.

Interestingly, some new singers were also able to find enjoyment in online singing. At the time of our conversation, one of my informants had only participated in stringing twice – her first encounter with Sacred Harp. She had a background in music and a great deal of experience with virtual communities, which she found fulfilling. This allowed her to feel deeply engaged with the activity and to connect with the other participants. I also encountered new singers at zingings. Most seemed to be seeking out community and musical participation, and saw Sacred Harp as an opportunity to access both. The fact that more experienced participants regularly shared memories and knowledge allowed relative newcomers (myself included) to quickly become familiar with the tradition, even in the absence of long experience.

The core of the Sacred Harp tradition is the songs themselves. It has been well-established that familiarity is key to the power of music to evoke an emotional response (Ali and Peynircioğlu, 2010, p. 181; Pereira et al., 2011; Schubert et al., 2014), and Sacred Harp singers are deeply committed to their shared repertoire. Some of my interlocutors spoke about the power of the repertoire to sustain community in the absence of the above-mentioned mechanisms. “We’re singing songs that connect us to one another,” I was told by a Pennsylvania singer. “And that sense of connection is above and beyond the specifics. It moves us into the general. So I do not need to worry about a real singing.” For her, the songs held absolute power to produce a meaningful community experience. A Minnesota singer described the same phenomenon, explaining that “online singings work,” despite their many shortcomings, “because [of] those songs, we are keeping those songs in our minds.” For these singers and others, online singings provided contact points with a communal repertoire that exists across time and place, and singing the songs granted access to a stream of memories (Jäncke, 2008; Belfi et al., 2016, p. 985). Although the act of singing had been reshaped by the pandemic, the songs and their attendant community – a community that is reinforced each time the songs are sung – remained untouched. It is also worth noting that the songs included in *The Sacred Harp* deal overwhelmingly with issues of mortality – a fact that for some made them particularly meaningful in the context of a global pandemic (Cobb, 1978, p. 24–25). It is difficult to sing a text like “Lord, while we see whole nations die, / Our flesh and sense repine and cry; / Must death forever rage and reign?/Or hast Thou made mankind in vain?” without being reminded of the devastation wrought by the pandemic (McGraw, 1991, p. 547).

#### Ritual

“Is it a cult?,” asked singer and scholar Buell Cobb of the Sacred Harp tradition in his 2014 memoir (xiii). Although he meant the remark to be tongue-in-cheek, there is much about the ritual of Sacred Harp and the sometimes-obsessive commitment of its participants that can appear cultish, and ritual has become a perennial focus of Sacred Harp studies (Heider and Warner, 2010). In discussing memory, I have already addressed many of the ritualistic aspects of Sacred Harp singing, for it is ritualized action that guides the accumulation of singing-related memories and gives them emotional weight (McCauley and Lawson, 2002, p. 38). I have also already described rituals related to the maintenance of local group identity, for ritual is the primary mechanism by which that identity is created and affirmed. The fact that facilitators of online singing explicitly chose to maintain rituals helped participants to feel a sense of communal continuity. “There’s this part of this that is absolute normalcy that I love,” commented a Minnesota singer. “It’s Tuesday night, I’m singing.” A Utah singer identified nearly the same motivation for engaging with online singing: “We’re doing this thing that we always do together, together.” For these singers and others, the opportunity to maintain the Sacred Harp habit outweighed any drawbacks of online singing.

Sacred Harp is characterized by cyclic ritual at all levels, including the annual parade of large in-person gatherings. Social media and the Sacred Harp listserv bore witness to the relentless string of cancelations that took place throughout 2020—announcements that never came as a surprise, but were always painful to organizers and habitual attendees. In a number of cases, however, annual events were reimagined online in ways that preserved their character and function, and many participants found these to be profoundly meaningful.

The 85th Annual Judge Jackson Singing became a brief zinging held on September 20. Although we only sang a single song, it was important to the organizer – a descendant of Jackson – to do something to mark the event, which is dedicated to upholding the legacy of *The Colored Sacred Harp*. This songbook, created by Jackson for use in the black community, is out of print and seldom used, and it is therefore of particular importance that rituals and memories associated with the volume be sustained.

*Southern Harmony* singers find themselves in a comparable situation. Although various groups use the book as an adjunct, only a single annual event is dedicated exclusively to *The Southern Harmony*: the Big Singing Day in Benton, KY. The cancellation of this event, which always takes place on the 4th Sunday in May, was a major blow to the community. However, it was reimagined as a May 24 stringing, with three singers broadcasting from Draffenville, KY, eight other singers live-overdubbing from other locations, and countless non-streaming participants singing along (Caudle, 2021). I spoke with one of the organizers, Erin Fulton, and she was astonished by the response from the community. “I did not understand what anybody was getting out of it,” she told me. “I did not feel good about the technological or musical success of what we wound up doing. But all of the responses that I got, both from people that I know well and from folks who had been interested in coming for a long time but were not able to go and from people who have roots in the area but have moved away and cannot go, that was really astounding.” In considering this response, she pointed out that the community of *Christian Harmony* singers is small and geographically-specific, and that there are few opportunities to hear these songs. She also described plans to stream the Big Singing in the future in order to increase access.

I also attended two annual Sacred Harp events that were reimagined as zingings: the Palo Alto Virtual “All-Day” Mid-Day Zinging (August 22) and the Minnesota Convention (September 26). The convention, in particular, was carefully designed to replicate and recall the in-person experience. Although the event was shortened to 2 h, the structure – opening prayer, singing, memorial lesson, business meeting, more singing – was retained. The organizers announced at the beginning that they had a playlist of recordings from past Minnesota conventions on hand in case there was a shortage of volunteers to call and live-lead songs, but in the end the recordings were not needed. Communal memory was certainly the theme of the gathering; a space was carved out between the memorial lessons and business meeting for participants to share their recollections of past conventions and singers, with a few invited speakers leading the way. “Live sharing is full of memories,” I wrote in my notes that day, “including the observation that songs remind us of people and evoke memories of their voices.”

In addition, singers found solace in the act of “showing up” for one another, committing to ritualized participation not just for themselves but to support the community. “I want to show up and participate for other people who are singing,” a Minnesota singer told me, “or who are feeling like we need to sing.” One of the zinging facilitators confirmed the value of “showing up” each week on behalf of her community: “The fact that I run it has made me feel like I have a purpose. I cannot do a lot in the world right now, but I can figure out how to use Zoom.” I observed a similar effect when studying participants in a virtual hymn-singing choir that was active in the early months of the pandemic (Morgan-Ellis, 2021a). For all of these singers, the value of participation in virtual singing was in part derived from the act of ritual service to others.

#### Learning and Growing

Personal musical development is – and always has been – at the heart of the Sacred Harp tradition. In the early 19th century, shape-note tune books were published and marketed by singing teachers, who made a living traveling the countryside to teach music-reading skills over the course of 2-week singing schools (Marini, 2003, p. 79). Volumes like *The Sacred Harp* were expressly designed for use in pedagogical settings and they included a range of repertoire that was intended simultaneously to satisfy the novice and challenge the expert (Cobb, 1978, p. 38). Much of the language surrounding Sacred Harp activity – weekly gathering are termed “practice singings,” a group of singers is called a “class,” and each individual leads a “lesson” – reflects this heritage, and singers still focus on teaching, learning, and improving their skills (Heider and Warner, 2010, p. 84).

While new singers are embraced by the community and invited to participate fully, “full” participation as theorized by scholars of communities of practice is predicated on deep knowledge of the songs and rituals. Many Sacred Harp singers seek to move from “peripheral” to “full” participation. A “full” participant in the community will probably be able to read music fluently and sing in an idiomatic style, and they might have the ability to “key” (give the starting pitches for) their own songs, although this is not universal. They will be conversant with the history of shape-note singing and know prominent singers. They will also be familiar with the entire volume and likely have many of the songs memorized. Seeing as there are 554 songs in the Denson revision alone, gaining this level of familiarity requires many years of intense dedication.

Almost all of my interlocutors expressed gratification at the learning opportunities afforded them by online singing, often focusing on the ways in which online singing was pedagogically superior to in-person singing. As one Minnesota singer told me, online singing “has pushed me even harder and faster” in terms of musical development. She counted herself among the many singers who found themselves forced to develop new skills in response to virtual environments. For example, singers who wanted to call songs in live-led zingings or anchor stringings needed the ability to key the songs and sing the tenor (or at least carry another part with confidence). At an in-person singing, there is always someone on hand to key a song if the leader does not want to do so, and it is not necessary to acquire the skill. “I would have never pitched my own song at this point, never,” confided the same Minnesota singer, who only has 2 years of experience. She had also been refining her ability to find the alto starting pitch without the aid of a section. Another Minnesota singer described how, although she usually sang treble, she was learning the tenor parts in order to lead. She was also learning to key, and credited zinging with pushing her to gain that skill: “I had been thinking about starting to pitch my own songs anyway and I had just never gotten to it in the regular singings because it was so hard to break through that barrier, and so now it’s an excuse to do it, and I’m getting better.” The singer in Scotland found herself needing to learn a new part in order to contribute to a virtual choir.

The incomplete textures of live-led zinging and stringing were appealing to some, who enjoyed the challenge of adding a missing part. This opportunity arises only in the most intimate in-person singings; usually, there are many confident singers in each section, and those still finding the notes are swept along with the tide. “I find it really fun to be the fourth part,” a singer who engages in stringing told me. “If you have people singing the other parts and you get to fill in the missing one, that’s very satisfying.” Although a California singer at first had trouble adding his part without support from a section, he found value in rising to meet the challenge. “I’ve found it difficult, but it’s a skill I’ve started to learn,” he told me. “It’s helping me to be more proficient, so that’s good.” Likewise, many singers appreciated the opportunity to really hear their own voices for the first time. “I can hear myself so much louder in the Zoom sing-alongs than I ever hear myself in an in-person singing,” reflected a Tennessee singer, “so I notice my own mistakes more and […] I feel like I’m learning some of the songs better and tighter because I’m hearing myself more loudly and clearly.”

Some found online singing to create new opportunities for experimentation and growth. Because zinging participants are muted, they are free to take musical risks. In a traditional singing, for example, a singer must choose the section in which they are going to sit and then sing only that part (or risk annoying their neighbors). Zinging participants, however, can switch parts on a whim – something that I and several of my interlocutors enjoyed doing. “If I’m muted, and nobody can hear me,” explained an informant, “then I do not have to sing the part I normally sing, and I’m not going to put anybody off.” Singers are also free to experiment with ornamentation or vocal production without fear of embarrassment. Others developed confidence by streaming over Facebook Live or making recordings in order to participate in virtual choirs. A Minnesota singer told me how she worked up the courage to contribute to the Easter Anthem video: “I was like, I can do this, I can do this, I can do this.” She had never recorded herself and felt daunted by the task, but was proud of herself for successfully rendering a solo performance.

Finally, many singers engaged in online activity in order to keep learning songs. One explained to me why zinging was superior to singing along with recordings in isolation: “Like a live singing, I do not know what other people are going to call.” If she were on her own, she would not be discovering and singing unfamiliar songs. Zinging participants frequently applauded visitors for introducing new songs to their group, or reminding them of songs they had forgotten. “That’s one thing that I’ve enjoyed with Zoom and the streaming,” remarked a Tennessee singer, “is that they’ll sing songs that I have not sung before.” A Utah singer agreed: “It’s been a nice, a very small silver lining to this to have a little more circulation with other groups.” The singer in Scotland also commented on how participation in video-based zingings allowed her to call songs she did not know well and wanted to learn. “If we are watching a video, I’m not having to worry about leading it, so I get to sing what I want to sing.” Other singers saw participation in zingings as facilitating their accumulation of new songs – an ongoing activity that did not need to be disrupted by the pandemic. One California singer told me how grateful she was to have the opportunity to deepen her knowledge of *The Shenandoah Harmony* through singing online: “It’s helpful to try to be learning some new songs.” For her and others, online singing was able to facilitate personal growth as a shape-note singer.

## Conclusion

Explanations for the success of virtual Sacred Harp singing can be found in the musical characteristics of the repertoire, the motivations for participation, and the nature of Sacred Harp community, which extends across time and place. Sacred Harp songs are, with few exceptions, pulse-driven and performed at a consistent, loud dynamic. There is no need for a conductor to guide phrasing, and singers can stay together without subtle communication. Although the melody is technically in the tenor, all of the parts tend to be equally “melodic” and interesting, and in many songs, all parts sing all the time. Because the harmonies are often incomplete and the voice-leading does not adhere to conventional practice, any part can be subtracted from the texture without causing undue damage. Such repertoire is ideally suited to online practices such as stringing and live-led zinging, and also simplifies participation in virtual choirs. Even more importantly, for Sacred Harp singers the act of singing constitutes the whole of the experience. Although individual singers value growth and improvement, there are no rehearsals and there is no performance. Instead, there is the ritualist practice of coming together as a community to sing a beloved repertoire, week after week and year after year. This process, which spans a lifetime, culminates only in the death and remembrance of individual members. When considered on a timescale such as this, it seems logical that a pandemic should fail to disrupt the Sacred Harp community, even as participants vividly mourn the loss of in-person singing.

However, this study is largely missing the voices of those who found virtual singing unrewarding, or who never had access in the first place. I set out to understand the experiences of regular participants, and I identified many ways in which online singing satisfied their needs for community, ritual, and personal growth. Unfortunately, I am not able to fully explain why other singers declined to participate, nor have I documented the emotional and psychological experiences of those who were suddenly and completely cut off from their singing communities. I hope that later studies will remedy these omissions, for not every Sacred Harp singer found meaning and solace in virtual participation.

As of publication, all of the activities described in this article are ongoing, and it seems likely that they will continue for at least the majority of 2021. Virtual Sacred Harp singing has attracted an increasing number of participants over the course of the pandemic, although it is hard to estimate the total number. Annual events have attracted over 100 people, while weekly zingings and stringings usually draw between 15 and 40. About 200 unique users have signed on to the Philadelphia Jamulus hub, and more singers continue to adopt the technology. It is clear that a large number of people have been impacted by the practices described above. These findings are also significant, however, because online singing will not end with the pandemic. Many of the groups that have been meeting online intend to carry on with virtual singing activity in some form. At the same time, the technologies that facilitate online music-making are improving – indeed, have improved since the drafting of this article – and more people are learning how to use them. The pandemic has kickstarted a process that was probably inevitable, and virtual music-making is here to stay.

## Data Availability Statement

The datasets presented in this article are not readily available because the recordings and transcriptions of the interviews can only be shared with IRB approval. Requests to access the datasets should be directed to esther.morgan-ellis@ung.edu.

## Ethics Statement

The studies involving human participants were reviewed and approved by the Institutional Review Board at the University of North Georgia. The patients/participants provided their written informed consent to participate in this study. Written informed consent was obtained from the individual(s) for the publication of any potentially identifiable images or data included in this article.

## Author Contributions

The author confirms being the sole contributor of this work and has approved it for publication.

### Conflict of Interest

The author declares that the research was conducted in the absence of any commercial or financial relationships that could be construed as a potential conflict of interest.
